# Bioabsorbable zinc ion induced biphasic cellular responses in vascular smooth muscle cells

**DOI:** 10.1038/srep26661

**Published:** 2016-06-01

**Authors:** Jun Ma, Nan Zhao, Donghui Zhu

**Affiliations:** 1 Department of Chemical, Biological and Bioengineering, North Carolina A&T State University, Greensboro, NC 27411, USA; 2 National Science Foundation (NSF)-Engineering Research Center for Revolutionizing Metallic Biomaterials, Greensboro, NC 27411, USA; 3 Department of Biomedical Engineering, Pennsylvania State University, State College, PA 16801, USA

## Abstract

Bioabsorbable metal zinc (Zn) is a promising new generation of implantable scaffold for cardiovascular and orthopedic applications. In cardiovascular stent applications, zinc ion (Zn^2+^) will be gradually released into the surrounding vascular tissues from such Zn-containing scaffolds after implantation. However, the interactions between vascular cells and Zn^2+^ are still largely unknown. We explored the short-term effects of extracellular Zn^2+^ on human smooth muscle cells (SMCs) up to 24 h, and an interesting biphasic effect of Zn^2+^ was observed. Lower concentrations (<80 μM) of Zn^2+^ had no adverse effects on cell viability but promoted cell adhesion, cell spreading, cell proliferation, cell migration, and enhanced the expression of F-actin and vinculin. Cells treated with such lower concentrations of Zn^2+^ displayed an elongated shape compared to controls without any treatment. In contrast, cells treated with higher Zn^2+^ concentrations (80–120 μM) had opposite cellular responses and behaviors. Gene expression profiles revealed that the most affected functional genes were related to angiogenesis, inflammation, cell adhesion, vessel tone, and platelet aggregation. Results indicated that Zn has interesting concentration-dependent biphasic effects on SMCs with low concentrations being beneficial to cellular functions.

Biodegradable metals, namely magnesium (Mg), zinc (Zn) and iron (Fe), represent the new generation of implantable medical scaffolds. Among them, Mg-based alloys have been widely explored in stent applications because of their biodegradability, bioabsorbility, and low thrombogenicity^1^,^2^,^3^,^4^. However, the main drawbacks of conventional Mg alloys are insufficient mechanical strength and rapid corrosion accompanied with hydrogen gas evolution, pH increase, and premature loss of mechanical integrity^5^. Previous studies on surface treatment^6^ and element alloying^7^ of Mg for stent application showed that these two methods enhanced the performance of Mg and its alloys at some degree but not sufficiently. More sophisticated efforts are in need to fully overcome such weaknesses of Mg materials. The outcomes of earlier clinical trials were also not ideal. The AMS INSIGHT trial showed that absorbable metal stent (AMS) did not demonstrate efficacy in the long-term patency over standard percutaneous transluminal angioplasty (PTA)^8^. Another PROGRESS-AMS trial revealed sound results immediately post implantation with diameter stenosis reducing from 61.5% to 12.6% and acute gain of 1.41 mm in diameter. Then after 4 months, diameter restenosis increased to 48.4%, indicating a negative remodeling occurred^9^.

Fe-based alloys are interesting candidates for stent application as their mechanical properties are similar to stainless steel^1^,^10^, a benchmark for stent materials. However, its low degradation rate cannot catch up with clinical needs and lead to similar reactions found in permanent implants^10^,^11^. Another concern is that the ferromagnetism of Fe-based alloys negatively affects the compatibility with certain imaging devices, such as magnetic resonance imaging (MRI)^10^.

Zn is an alternative to Mg and Fe (or perhaps a better choice) for cardiovascular stent application after all because of its better mechanical and corrosion properties. In fact, Zn has been used as alloying element for Mg to enhance corrosion resistance^12^, increase strength and ductility simultaneously^13^, and decrease hydrogen evolution^14^. Because of its advantageous roles in alloy development and human nutrition, Zn was explored as stent implant in a rat model. The degradation rate was slow and ~70% cross section area remained within first 4 months, indicating the stably maintained mechanical integrity during healing process^15^. The follow-up study by the same group revealed low cell densities, low neointimal tissue thickness, and tissue regeneration within the corroding Zn implant. After 6.5 months, there was no neointimal tissue thickness progression. No obvious SMCs growth around the implant was observed during the entire course of experiment. It demonstrated that Zn might suppress the SMCs activities^16^ which is closely related to restenosis. *In vitro* study showed that pure Zn had lower corrosion rate, better hemocompatibility, and low cytotoxicity for cardiovascular stent application, compared to high purity Mg^17^. Zn alloying with other elements, such as Mg, Ca, Sr, and Mn were also explored regarding mechanical strength, corrosion resistance, biocompatibility, and hemocompatibility with encouraging outcome^11^,^18^,^19^,^20^. These studies demonstrated the potential of Zn as promising material for cardiovascular stent application.

Restenosis is one of the main problems reported in stent application^21^. SMCs migration, proliferation and excessive extracellular matrix (ECM) deposition, are responsible for restenosis after stent implantation^21^. Therefore, understanding of how Zn^2+^ affect the cellular behaviors of SMCs, especially cell proliferation and cell migration, could provide useful information on the mechanism of restenosis for Zn-based stent. In this study, we evaluated the effects of extracellular Zn^2+^ on cellular behaviors of SMCs in a short-term fashion up to 24 h, including cell viability, cell proliferation, cell adhesion, cell spreading, cell migration, cytoskeleton reorganization and cell morphology. Moreover, we also explored the differential gene expression changes of SMCs treated with Zn^2+^, which is the root cause of the cellular behavior changes.

## Materials and Methods

### Zn^2+^ solutions preparation

ZnCl_2_ solution was prepared by dissolving ZnCl_2_ (Sigma Aldrich, US) in deionized water. The solution was filtered by 0.22 μm filter (Fisher Scientific, US) and autoclaved (Harvey Sterile Max, Thermo Scientific, US). The ZnCl_2_ solution was diluted by SMC medium (SMCM, ScienCell, US) into different concentrations. The pH values of diluted solutions were measured by pH meter (Fisher Science Education, Fisher Scientific, US).

### Cell culture

Human aorta smooth muscle cell (HASMC, ScienCell, US) was expanded in 75 cm^2^ culture flask (Falcon, BD Biosciences, US). The culture medium was SMCM with 2% fetal bovine serum (FBS, ScienCell, US), 1% SMC growth supplement (SMCGS, ScienCell, US) and 1% penicillin/streptomycin solution (P/S, ScienCell, US). When cells reached 90% confluence, they were washed by Dulbecco’s phosphate-buffered saline (DPBS, Life technologies, US) and then detached by trypsin/EDTA solution (Life technologies, US). After detachment, SMCM was added and the cell solution was centrifuged (Sorvall Biofuge Stratos, Thermo Electron Corporation, US). The supernatant was removed and cell pellet was resuspended by SMCM. The cell solution was mixed with trypan blue stain (Life technologies, US) and cell number was counted by hemocytometer (Bright-Line, Hausser Scientific, US) under microscope (EVOS FL Cell Imaging System, AMG, US). Then cell solution was diluted into different densities for the following tests.

### Cell viability test

Cell viability was detected by 3-(4,5-dimethylthiazol-2-yl)-2,5-diphenyltetrazolium bromide (MTT, Life technologies, US). SMCs were seeded into a 96-well plate (Falcon, Corning, US) at density of 5,000 cells/well 100 μL and incubated for 24 h. Cell medium was replaced by different Zn^2+^ solutions and incubated for 24 h. After that, solutions were replaced by 100 μL fresh medium supplemented with 10 μL of 12 mM MTT stock solution and incubated for 4 h. Then 100 μL SDS-HCl solutions were added to each well and incubated for another 4 h. The absorbance was measured by microplate reader (Molecular Devices, US) at 570 nm. SMCM with and without cells were used as positive and negative control, respectively. Cell viability was determined as below:


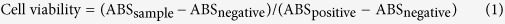


### Cell proliferation test

Cell proliferation was measured by Bromodeoxyuridine assay (BrdU assay, Cell Signaling, US). Cells were seeded into a 96-well plate at density of 5,000 cells/well 100 μL and allowed to attach for 24 h. Then cell culture medium was replaced by of Zn^2+^ solutions with different concentrations and cells were incubated for 24 h. After that, the following steps were performed, following the manufacturer’s protocol. Briefly, solutions were replaced by culture medium supplemented with 1X BrdU buffer and incubated for 3 h at 37 °C. After that, solutions were removed and 100 μl fixing/denaturing solutions were added to each well. The plate was kept at room temperature for 30 min and solutions were removed. Then 100 μl 1X detection antibody solution was added to each well and the plate was kept at room temperature for 1 h. Solutions were removed and plate was washed by 1X Wash Buffer for three times. Then 100 μl 1X HRP-conjugated secondary antibody solutions were added to each well and the plate was kept at room temperature for 30 min. The solutions were removed and plate was washed three times by 1X Wash Buffer. Then 100 μl of TMB substrate was added to each well and incubated for 30 min at room temperature. After that, 100 μl stop solution was added. Finally, absorbance was measured by microplate reader (Molecular Devices, US) at 450 nm. Cell proliferation was calculated by the formula below:


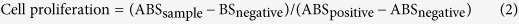


### Cell adhesion test and centrifugation assay

SMCs were mixed with Zn^2+^ solutions and seeded into a 24-well plate (Falcon, Corning, US). The final cell density was 50,000 cells/well. The cells were allowed to attach for 2 h. Then medium was removed and cells were washed by DPBS. The images of adhered cells were taken by microscope (EVOS FL Cell Imaging System, AMG, US) and the plate was sealed by self-sticking tape (Fisher Scientific, US). The plate was inversely put into the rotor and centrifuged at 1000 rpm for 5 min. Cells were washed by DPBS and fixed by 4% paraformaldehyde (ChemCruz, Santa Cruz Biotechnology, US). Images of attached cells were taken by microscope and analyzed by Image J (NIH, US). At least 10 different fields were used for calculating attached cell density and cell retention ratio.


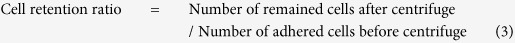


### Cell migration test

SMCs were seeded into a 24-well plate and after a monolayer was formed, cell medium was removed. A P200 pipette tip (Thermo Scientific, US) was used to create a scratch. Dead cells and cell debris were washed away by DPBS. Cells were treated with Zn^2+^ solutions for 6 h. At 0 h and 6 h, images of the scratch was taken by microscope (EVOS^®^ FL Cell Imaging System, AMG, US) and analyzed by Image J (NIH, US). At least 10 different points were chose to determine width of the scratch for each concentration group. Because cell migrated toward the scratch from two directions simultaneously, half of the width change was used to calculate average migration rate of cells.


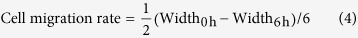


### Cell spreading

SMCs were mixed with 40 μM and 120 μM Zn^2+^ solutions and seeded into 24-well plates at density of 50,000 cells/well. At 0 h, 2 h, 4 h, 6 h and 8 h, cells were stained by calcein AM (Life technologies, US) and images were taken by microscope (EVOS FL Cell Imaging System, AMG, US). Images were analyzed by Image J (NIH, US). In order to determine cell area and cell perimeter, at least 30 cells were chose for each concentration group.

### Cytoskeleton staining and cell morphology

Cells were seeded onto cover glasses (Fisher Scientific, US) at a 24-well plate at density of 50,000 cells/well and incubated for 24 h. Cell medium was replaced by Zn^2+^ solutions and incubated for another 24 h. After that, solutions were removed and cells were washed by DPBS. Cells were fixed by 4% paraformaldehyde (ChemCruz, Santa Cruz Biotechnology, US) and permeabilized by 0.1% Triton X-100 (Thermo Scientific, US). Solution was removed and cells were washed by DPBS. Cells were blocked by 5% bovine serum albumin (BSA, Fisher Scientific, US) for 1 h. Solution was removed and cells were washed by DPBS. 2 μg/ml recombinant rabbit monoclonal vinculin antibody solution (Life technologies, US) was added to each well and incubated for 2 h at room temperature. The solution was removed and cells were washed by DPBS. After that, 4 μg/ml Alexa Fluor 546 goat-anti-rabbit IgG solution (Life technologies, US) was added and incubated for 2 h at room temperature in dark. Cells were washed by DPBS and one drop of Actin Green 488 Ready Probes Reagent (Life technologies, US) was added onto the cover glass. The plate was kept at room temperature in dark for 30 min. After that, one drop of ProLong Gold Antifade Mountant with DAPI (Life technologies, US) was added to a clean glass slide (Thermo Scientific, US) and the cover glass was inversely put on the glass slide. The cover glass was sealed by CoverGrip Coverslip Sealant (Biotium, US). The images of immunostaining were taken by fluorescent microscope (EVOS FL Cell Imaging System, AMG, US) and analyzed by ImageJ (NIH, US). At least 30 cells were used for morphology characterization. Cell aspect ratio and cell circularity were determined as below:


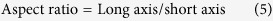



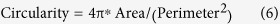


### Gene expression profiles

Cells were seeded into 60 mm petri dishes (Primaria, Corning, US) and after a monolayer was formed, cell medium was replaced by SMCM supplemented with 40 μM or 120 μM Zn^2+^ solutions. After incubation for 24 h, total RNA was isolated by RNeasy Mini Kit (Qiagen, US). The concentration and purity of total RNA were determined by spectrophotometer (Nanodrop 2000, US) at 230 nm, 260 nm and 280 nm. The range of A_260_/A_280_ was 2.00–2.05 and the range of A_260_/A_230_ was 2.20–2.29. Then 500 ng total RNA was inversely transcripted into cDNA by RT^2^ First Strand Kit (Qiagen, US), according to the manufacturer’s protocol. RT-PCR was performed in a CFX96 Touch RT-PCR Detection System (Bio-Rad, US) using microarray. cDNA was mixed with RT^2^ SYBR Green Master Mix (Qiagen, US) and RNase-free water. In the 96-well PCR array plate, 25 μl of the mixture was added to each well. After 10 min incubation at 95 °C, cDNA was amplified according to the following parameters: denaturing for 15 s at 95 °C, then annealing for 1 min at 60 °C. 40 cycles were performed. After the amplification, Ct values were collected and analyzed by Bio-Rad CFX Manager 3.1 (Bio-Rad, US). The cutoff Ct value was 35. The ΔΔCt method was used to calculate the fold change.

### Statistical analyses

All data were presented as mean ± standard deviation and at least three replicates were used in each test for each concentration group. Student’s t-test was used in statistical analyses (Prism 5, GraphPad Software, US) and *p* < 0.05 was considered significant.

## Results

### High concentration of Zn^2+^ inhibited cell viability

**Figure 1:**
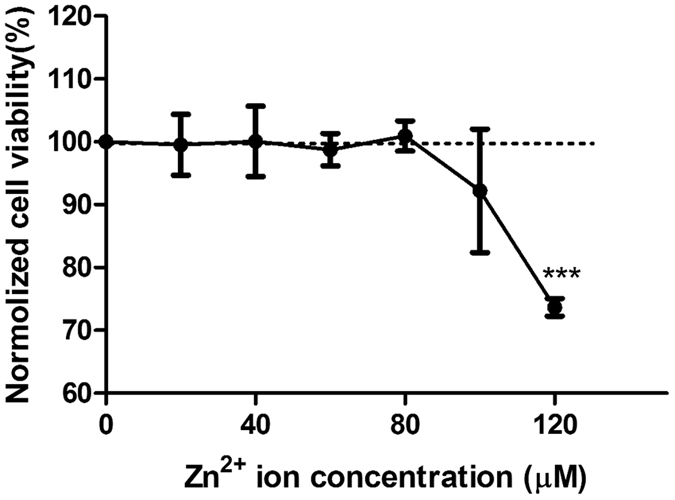
Cell viability of SMCs treated with Zn^2+^ with different concentrations for 24 h. Student’s t-test, **p < 0.01, ***p < 0.001.

When below 80 μM, Zn^2+^ had no adverse effects on cell viability but above 100 μM, Zn^2+^ inhibited cell viability and significance was observed when concentration reached 120 μM (p < 0.001) (Fig. 1). In addition, pH in diluted Zn^2+^ solutions had no obvious changes (data not shown).

### Zn^2+^ had biphasic effects on cell proliferation

**Figure 2:**
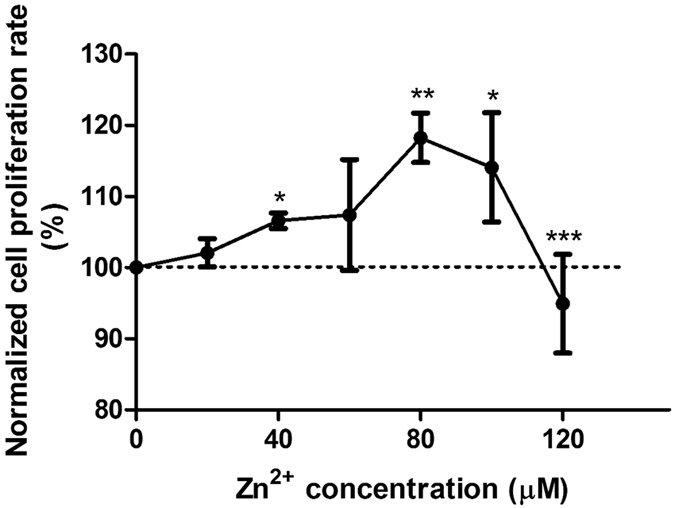
Cell proliferation of SMCs cultured with different Zn^2+^ solutions for 24 h. Student’s t-test, **p < 0.01, ***p < 0.001.

As shown in Fig. 2, Zn^2+^ had a bell-shape biphasic effect on cell proliferation. Zn^2+^ promoted cell proliferation with increasing concentration and reached maximum around 80 μM, and then proliferation started to decrease with further increasing of Zn^2+^ concentration. For all concentrations up to 110 μM, the proliferation profile remained above base line (100%). However, Zn^2+^ inhibited cell proliferation significantly when it was above 120 μM (p < 0.001).

### Zn^2+^ altered cell adhesion and cell adhesion strength in dose-dependent manners

**Figure 3:**
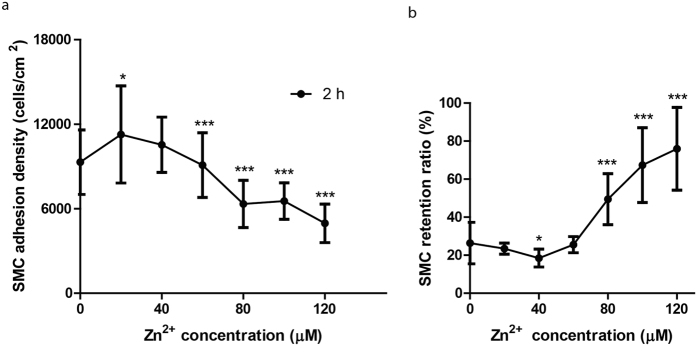
SMC adhesion at 2 h (**a**) and the cell retention ratio after centrifuge (b). Student t-test, **p < 0.01, ***p < 0.001.

Cell adhesion was in a Zn^2+^ concentration-dependent manner. Cells were allowed 2 h to attach. Zn^2+^ increased cell adhesion density when below 40 μM while inhibited cell adhesion when above 40 μM (Fig. 3a). Interestingly, Zn^2+^ had an opposite effect on cell retention compared to cell adhesion. After centrifuge, lower percentage of cells remained attached when treated with lower concentration of Zn^2+^ (0–40 μM) while higher percentage of cells remained attached for higher concentration (80–120 μM) (Fig. 3b).

### Zn^2+^ induced biphasic changes on cell spreading

**Figure 4:**
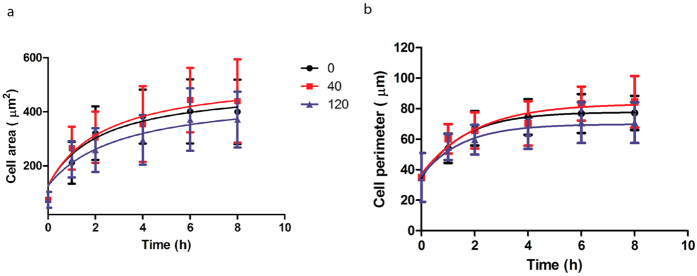
SMC spreading up to 8 h. (**a**) cell area; (**b**) cell perimeter. Student’s t-test, **p < 0.01, ***p < 0.001.

Cell spreading was examined with one typical low (40 μM) and one typical high (120 μM) concentrations of Zn^2+^ for up to 8 h (Fig. 4). At 40 μM, Zn^2+^ promoted cell spreading and cells had a larger cell area and perimeter. In contrast, at 120 μM, Zn^2+^ inhibited cell spreading and cells tended to have smaller cell area and perimeter. For all experimental groups, cell spreading reached a plateau phase after 8 h.

### Zn^2+^ had biphasic effects on cell migration

**Figure 5:**
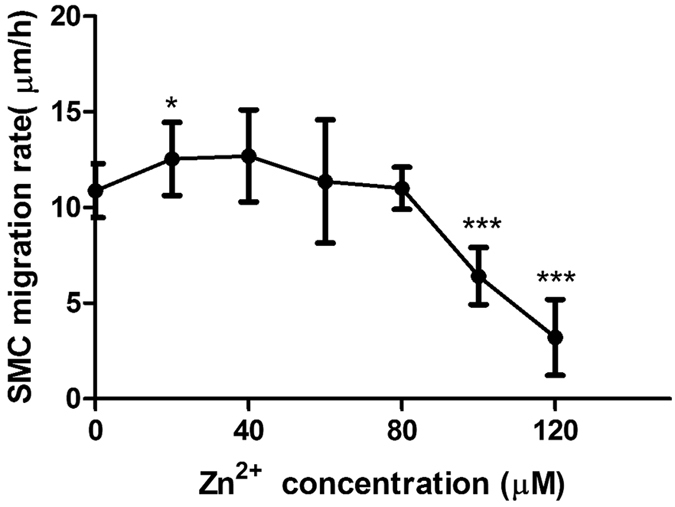
The average migration rate of SMCs treated with Zn^2+^ for 6 h. Student’s t-test, **p < 0.01, ***p < 0.001.

Similar to cell proliferation data, cell migration rate had a bell-shape relationship with Zn^2+^ concentrations (Fig. 5). Zn^2+^ increased cell migration rate when below 80 μM while decreased cell migration rate significantly when above 100 μM (p < 0.001).

### Effects of Zn^2+^ on cytoskeleton reorganization and cell morphology

**Figure 6:**
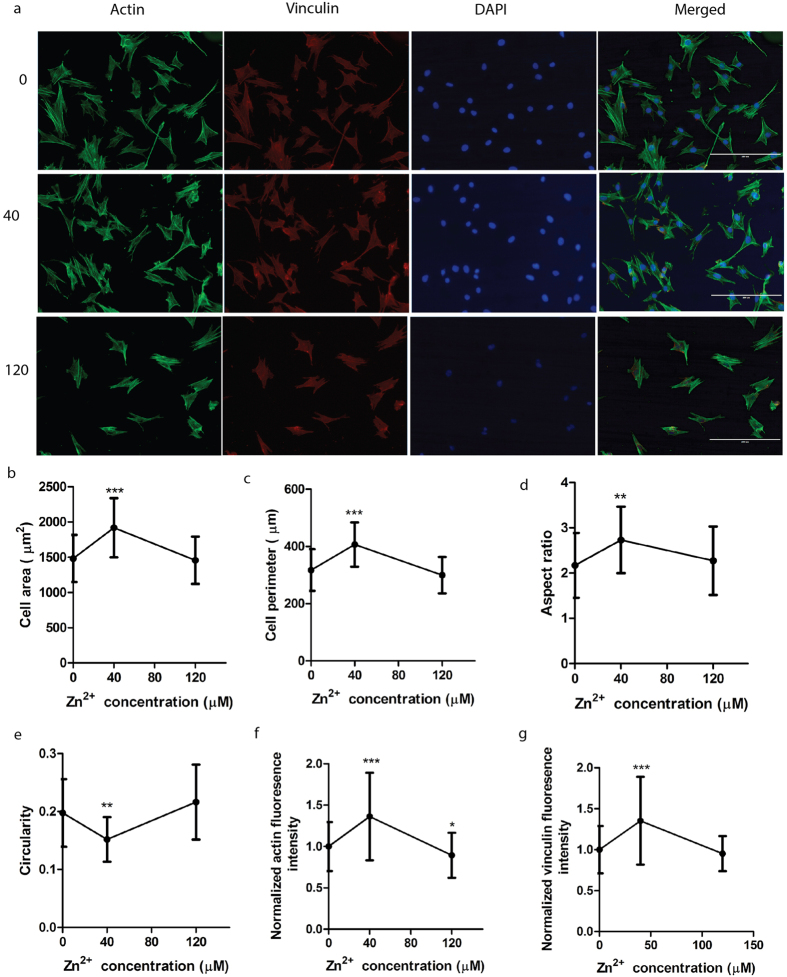
Effects of Zn^2+^ on cytoskeleton and cell morphology. (**a**) Representative images of cytoskeleton of SMC after treatment by Zn^2+^ for 24 h; (**b**) cell area; (**c**) cell perimeter (**d**) cell aspect; (**e**) cell circularity; (**f**) normalized expression of actin; (**g**) normalized expression of vinculin. The scale bar is 200 μm. Student’s t-test, **p < 0.01, ***p < 0.001.

Similar to previous cell spreading test, cytoskeleton reorganization and cell morphology were examined with one typical low (40 μM) and one typical high (120 μM) concentrations of Zn^2+^. The representative images were shown in Fig. 6a. Compared to control, cells treated with 40 μM Zn^2+^ had larger cell area and perimeter (Fig. 6b,c). In contrast, cells treated with 120 μM Zn^2+^ tended to have smaller area and perimeter (Fig. 6b,c). Cell morphology was characterized by aspect ratio and circularity. Cells were likely to display an elongated shape when treated with 40 μM Zn^2+^. Cells displayed a more round shape when treated with 120 μM Zn^2+^ compared to controls without any treatment (Fig. 6d,e). Actin and vinculin expression were enhanced at 40 μM Zn^2+^ but inhibited at 120 μM Zn^2+^ (Fig. 6f,g).

### Zn^2+^ regulated gene expression profiles differentially

**Figure 7:**
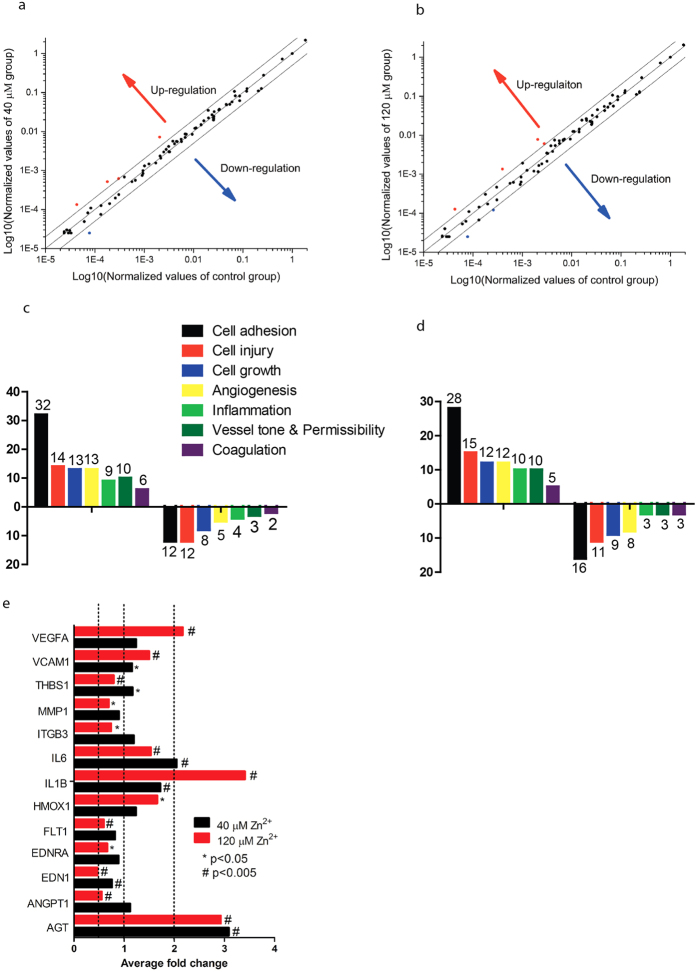
Effects of Zn^2+^ on SMCs gene expression profiles. (**a**) The scatter plot of gene expression profiles of SMCs treated with 40 μM Zn^2+^; (**b**) The scatter plot of gene expression profiles of SMCs treated with 120 μM Zn^2+^; (**c**) Number of functional genes regulated at 40 μM Zn^2+^; (**d**) Number of functional genes regulated 120 μM Zn^2+^; (**e**) Genes were significantly regulated at 40 μM Zn^2+^ or 120 μM Zn^2+^.

Zn^2+^ changed the gene expression profile of SMCs significantly. With 40 μM Zn^2+^ treatment, four genes were up-regulated and one gene was down-regulated by at least two-fold (Fig. 7a). With 120 μM Zn^2+^ treatment, four genes were up-regulated and two genes were down-regulated by at least two-fold (Fig. 7b). The most affected functional genes were related to cell adhesion, cell injury, cell growth, angiogenesis, inflammation, vessel tone, and coagulation (Fig. 7c,d). Among them, genes *AGT, ANGPT1, EDN1, FLT1, ILB1, IL6, ITGB3, MMP1, THBS1, VCAM1*, and *VEGFA* were significantly regulated by at least two-fold (Fig. 7e).

## Discussion

Zn is a promising biodegradable metal for cardiovascular stent applications due to its good mechanical and corrosion properties as well as biocompatibility. However, there is very limited study on how Zn may affect the vascular cell behaviors, especially SMCs which is the major player in stent restenosis and thrombogenesis. Endothelial cells and SMCs are the main cellular components of the vessel tissue. They play important roles in growth and maintenance of physiological functions and normal structure of vessel wall, as well as in vascular diseases^22^,^23^. In cardiovascular stent application, endothelial cell proliferation and migration affect re-endothelialization, which is an important factor to evaluate the overall performance of stent materials^4^,^24^. After stent implantation, rapid re-endothelialization on the stent surface are essential in preventing intimal thickening and vascular thrombosis^25^. In contrast, proliferation and migration of SMCs and extracellular matrix deposition contribute to restenosis^26^. Such different cellular behaviors and outcome from endothelial cell and SMCs led us to hypothesize that Zn ion may have different effects on these two types of vascular cells. Here, we explored the interesting effects of Zn ion on SMCs as a continuous investigation of Zn and vascular interactions^25^.

Understanding the cellular behaviors of endothelial cells and SMCs exposed to surrounding microenvironment are critical in stent application^25^,^27^. Intense policy regulation on implantable medical device would require such information to be fully disclosed for any Zn-based device. Therefore, it is essential to understand the interactions between the local vascular cells and Zn ion at the cellular and molecular levels. Here, we reported for the first time that how Zn ion changes the cellular behaviors of SMCs *in vitro* in a short term.

Zn^2+^ altered SMCs viability and proliferation in a dose-dependent manner. Lower concentrations of Zn^2+^ had no obvious effects on viability up to 80 μM Zn^2+^ but significantly inhibited cell viability and proliferation at 120 μM. This is in line with a previous study that Zn^2+^ had no effect on cell viability of human airway SMCs if below ~77 μM and decreased cell viability significantly if above ~115 μM^28^. However, different SMCs from different species may respond differently. For example, at ~38 μM, Zn^2+^ significantly inhibited proliferation of SMCs from carotid artery of Wistar rat. In a carotid artery injury model, injection of 5 mg/kg ZnCl_2_ for 14 days significantly inhibited neointimal formation and increased lumen area slightly^29^. SMCs from tracheas and bronchi of Brown Norway rat had a significantly higher proliferation with a 3–24 μM concentration range of Zn^2+^, and cell proliferation was significantly inhibited by Zn^2+^ at 96 μM^30^. In addition, SMCs from different tissue origin in the same species may have different responses. Human SMCs from benign prostatic hyperplasia had higher Zn^2+^ tolerance compared to human coronary SMCs. Zn^2+^ promoted prostatic SMCs proliferation with a range of 50–250 μM while significantly inhibited their proliferation when above 250 μM. The underline molecular signaling pathway for such biphasic effects of Zn on SMCs could be a complex. The activation of mitogen-activated protein kinases (MAPKs) and phosphoinositol 3-kinase (PI3K) pathways by Zn^2+^ may count for the enhanced cell proliferation including human bronchial epithelial cell^31^ and Swiss 3T3 cell^32^. MAPK pathway was also involved in a biphasic relationship between Zn^2+^ and HT-29 colorectal cancer cell growth^33^. Treatment with 10 μM Zn^2+^ activated ERK transiently and induced cell cycle regulator cyclin D1 while 100 μM of Zn^2+^ induced prolonged ERK activities and the increase of cyclin D1 and p21^Cip/WAF1 ^33.

Cell adhesion and spreading were also largely dependent on Zn^2+^ concentrations. The threshold concentration of Zn^2+^ for promoting or inhibiting SMCs adhesion was ~40 μM in this study. The cell-substrate adhesion involves proteins in ECM, transmembrane receptors and cytoskeleton. Transmembrane receptors and cytoskeleton serve as local anchorage and link cells to ECM^34^. Of the genes responsible for SMC-substrate adhesion examined, only *ITGB3* was significantly inhibited at 120 μM of Zn^2+^. Additionally, F-actin and vinculin expression were enhanced significantly at 40 μM Zn^2+^ while decreased at 120 μM. Previous study revealed that vinculin could promote cell spreading by stabilizing focal adhesions and transferring mechanical stresses that drive cytoskeletal remodeling^35^. This result was consistent with our observation that overexpressed vinculin was accompanied by the promoted cell spreading at 40 μM Zn^2+^. Moreover, cell spreading is tightly related to cell proliferation. Cell spreading is often accompanied by changes in the structure and composition of the cytoskeleton and distinctive changes in cell behaviors, such as growth and dedifferentiation^36^. Study showed that restriction of SMC spreading in one direction changed cell morphology and decreased cell proliferation^37^. When cell spreading was confined within a small cell area ranging 300–500 μm^2^ on a micropatterned matrix, cell proliferation decreased, probably because of a limited cell spreading^38^. In consistent with previous studies, cell area was within a small range of 200–400 μm^2^ when cell spreading reached a plateau phase and decreased cell proliferation was accompanied with decreased cell area and perimeter in this study. The inhibitory effects of confined cell spreading on cell growth was probably because the limited spreading reduced the intracellular pH^39^. Zn^2+^ could activate Na^+^/H^+^ exchanger 1 (NHE1) by binding to an extracellular zinc-sensing receptor (ZnR) and triggering the release of calcium ion (Ca^2+^) that subsequently activates extracellular signal-regulated protein kinases 1 and 2 (ERK1/2) and MAPK pathway^40^. The confined SMC spreading accompanied with decreased cell proliferation at higher concentration of Zn^2+^ was probably due to the deactivation of NHE1 by higher concentration of Zn^2+^, leading to a slight decrease in intracellular pH and thereby inhibiting cell growth.

Cell morphology changes requires the transfer of mechanical forces between the cytoskeleton and ECM as well as alterations in cytoskeletal organization^35^. It is well recognized that cell morphology is tightly coupled to DNA synthesis and cell growth^41^. We found that lower concentrations of Zn^2+^ promoted DNA synthesis and converted SMC from a relatively round shape to a spindle shape. The relatively elongated shape resembles the morphology of SMC *in vivo* under physiological condition^42^, which might indicate the beneficial effects of lower concentrations of Zn^2+^.

SMC migration, proliferation and excessive ECM deposition contributes to restenosis after stent implantation^21^. In this study, we found lower concentrations of Zn^2+^ promoted cell migration while higher concentrations of Zn^2+^ inhibited it. To remain motile, a cell must maintain a low level of adhesion to the extracellular matrix to allow traction^43^. Centrifugation assay showed that lower concentrations of Zn^2+^ had a lower cell retention ratio and high concentrations of Zn^2+^ increased cell retention ratio. These data indicated that cells treated with lower concentrations of Zn^2+^ had a lower level of adhesion strength with less firm attachment. This observation might explain the corresponding biphasic effects of Zn^2+^ on cell migration.

Finally, we investigated the gene expression profiles of SMCs because they are the ultimate root causes for any observed cellular behavior in most cases. Gene expression data revealed that the most affected functions were angiogenesis, inflammation, cell adhesion (cell-cell adhesion and cell-substrate adhesion), vessel tone, and platelet aggregation. For on particular cell function change, there might be several genes involved in with different levels of regulation. For example, *FLT1* gene, encoding vascular endothelial growth factor receptor 1, was significantly down-regulated at 120 μM of Zn^2+^, whereas another gene *VEGFA*, encoding vascular endothelial cell growth factor A, was significantly up-regulated. These two genes were both involved in angiogenesis but regulated differentially by the same concentration of Zn^2+^. The overall phenotype, therefore, is a combination of different genes regulated at different levels.

Despite some similarities in trend of cellular responses from endothelial cells and SMCs to Zn ions, there are significant differences. Although both endothelial cells and SMCs have biphasic cellular behaviors with exposure to Zn ions, one of the most notable differences was the threshold concentration for specific cellular behavior^25^. Within the same concentration range, it is of great interest to compare the tolerance of endothelial cells and SMCs to Zn ions for cardiovascular stent application. We found that endothelial cells had higher tolerance of Zn ions than SMCs within 0–140 μM even though there were some variances for cell proliferation, which is consistent with recent studies^44^,^45^. Since endothelial cells directly contact with stent surface, a higher Zn tolerance could be beneficial for re-endothelialization with minimal chance of restenosis. *In vivo* study also showed that progression of neointimal tissue was checked and the activities of SMCs might be suppressed by the corrosion products of Zn^16^. It was likely that the concentration of Zn ions around the implant may exceed the cytotoxicity threshold concentration for SMCs, which provided a reasonable explanation for the inhibition of SMCs by Zn implants. Taken together, in the future designing of Zn alloys for stents, it would be beneficial to control the local concentration of Zn ion from implant degradation above cytotoxicity threshold for SMCs while below that of endothelial cells while the corrosion rate and mechanical properties should still match the healing phases of injured vessels^46^.

In conclusion, lower concentrations (<80 μM) of Zn^2+^ had no adverse effects or beneficial effects on SMC adhesion, spreading, viability, proliferation, and migration. Moreover, lower concentrations of Zn^2+^ enhanced the expression of actin and vinculin and cells displayed an elongated shape. Gene expression profiles showed that significantly affected genes were related to angiogenesis, inflammation, cell adhesion, vessel tone, and platelet aggregation. Results revealed that the biocompatibility and cellular behaviors were tightly related to Zn^2+^ concentrations. Therefore, this study could provide useful information for a better stent design. A controlled release of Zn ion from Zn-based stent through controlled degradation pace could regulate SMC behaviors and the occurrence of restenosis.

## Additional Information

**How to cite this article**: Ma, J. *et al*. Bioabsorbable zinc ion induced biphasic cellular responses in vascular smooth muscle cells. *Sci. Rep.* **6**, 26661; doi: 10.1038/srep26661 (2016).
